# *Rhodococcus equi* Joint Sepsis and Osteomyelitis Is Associated With a Grave Prognosis in Foals

**DOI:** 10.3389/fvets.2019.00503

**Published:** 2020-01-14

**Authors:** Nicholas A. Ruocco, Lauren K. Luedke, Lisa A. Fortier, Norm G. Ducharme, Heidi L. Reesink

**Affiliations:** Department of Clinical Sciences, Cornell University College of Veterinary Medicine, Ithaca, NY, United States

**Keywords:** septic arthritis, septic physitis, orthopedic infection, bone abscess, *R. equi*

## Abstract

The most common pathologic manifestation of *Rhodococcus equi*, a gram-positive, facultative intracellular bacterium, is pyogranulomatous pneumonia in foals and weanlings. Hematogenous spread of bacteria may subsequently occur, resulting in joint sepsis, osteomyelitis, or subcutaneous abscessation. Medical records from horses presenting to the Cornell University Equine Hospital from 1998 to 2018 were reviewed for cases diagnosed with *R. equi* joint and/or bone infection, and information about case progression and outcome were analyzed. We hypothesized that, despite advances in diagnostic imaging, antimicrobials and antimicrobial delivery methods, the prognosis for *R. equi* joint sepsis and osteomyelitis remains grave for athletic activity and poor for survival. The 12 cases that met the review criteria had a mortality rate of 84% (10/12), with one case lost to follow up after discharge and one case discharged with a grave prognosis for athleticism.

## Introduction

Orthopedic infections caused by *Rhodococcus equi (R. equi)* can be difficult to diagnose and challenging to treat (1). Infection with *R. equi*, a gram-positive, facultative intracellular bacterium, typically presents as pyogranulomatous pneumonia in 1- to 6-month old foals. A common co-morbidity associated with *R. equi* pneumonia is hematogenous spread of bacteria (2), which can manifest as joint sepsis, vertebral body osteomyelitis or subcutaneous abscessation (3). The mortality rate for foals with clinical signs associated with *R. equi* pneumonia has been reported to be 30% (4), with mortality rates as high as 57% in foals with *R. equi* pneumonia in combination with extra-pulmonary disease (5, 6). Foals with septic arthritis have a mortality rate of 22–58% independent of bacterial isolate (7).

Joint sepsis secondary to hematogenous dissemination of bacteria is common in foals, and mortality rates for infection with various pathogens (e.g., *Enterobacteriaceae, Salmonella, Actinobacillus equuli, Klebsiella* spp., *Staphylococcus, Streptococcus*, and *R. equi*, etc.) are reported to range from 42 to 84% (2). The prognosis for osteomyelitis caused by *R. equi* has been described as guarded to grave; however, many of these reports are more than 20 years old (3, 8, 9). Therefore, the objective of this case series was to review treatment regimens and outcomes for cases presenting to a tertiary referral hospital for *R. equi* orthopedic infection. We hypothesized that, despite advances in diagnostic imaging, antimicrobials and antimicrobial delivery methods, the prognosis for *R. equi* joint sepsis and osteomyelitis remains poor.

## Materials and Methods

Medical records from horses presenting to the Cornell University Equine Hospital from 1998 to 2018 were reviewed with a text-based search engine using the keywords: *Rhodococcus equi*, foal, joint sepsis or osteomyelitis. This search yielded all discharge statements with at least one of the four keywords. Of those results (>10,000), only cases (10) that met the following criteria were included: (1) horses < 1 year of age and (2) diagnosis of a septic joint and/or osteomyelitis with a concurrent positive *R. equi* synovial fluid or bone culture or thoracic radiographic changes consistent with *R. equi* infection. Date of hospitalization, age, breed, location or joint(s) infected, degree of lameness based on the American Association of Equine Practitioners (AAEP) lameness scale, ancillary diagnostics, treatments, antimicrobial usage, duration of treatment and outcomes were recorded (Table 1). Owner written consent was obtained for publication.

**Table 1:** Case data from foals with confirmed *R. equi* joint and/or bone sepsis from 1998 to 2018.

**Date**	**Age**	**Breed**	**Location/joint**	**Antimicrobial therapy**	**Positive *R. equi* culture samples**	**Duration of treatment at CUHA**	**Outcome & necropsy findings**
1998	240 days	Arabian	Scapular osteomyelitis/ abscess	Chloramphenicol (PO), Clarithromycin (PO), Erythromycin (PO), Metronidazole (PO), Rifampin (PO), TMS (PO), Amikacin (IA)	Bone and subcutaneous abscess samples	14 days	Euthanasia
1998	365 days	Arabian	Cubital joint	No antimicrobial therapy—owner elected euthanasia	Synovial fluid and wound samples	1 day	Euthanasia. Locally extensive, severe, chronic purulent osteomyelitis of left antebrachium
2001	60 days	TB	Coxofemoral joint	Erythromycin (PO), Rifampin (PO) Amikacin (IA), Erythromycin (IA)	Synovial fluid; trans-tracheal wash	12 days	Euthanasia. Left coxofemoral joint: severe, chronic, ulcerative arthritis with severe bone lysis
2001	105 days	TB	Stifle joint	Erythromycin (PO), Gentamicin (IV), Rifampin (PO)	Femoral bone sample	5 days	Euthanasia
2002	45 days	TB	Coffin joint	Azithromycin (PO), Gentamicin (IV), Potassium Penicillin (IV), Amikacin (IA)	Synovial fluid: gram positive cocci; lung abscesses on radiographs	15 days	Discharged
2002	60 days	TB	Bilateral tarsocrural joints	Azithromycin (PO), Ceftiofur (IV), Rifampin (PO)	Trans-tracheal wash: gram positive pleomorphic rods; bone and joint samples culture negative	2 days	Euthanasia. Bilateral hock and stifle joints: mild, multifocal, fibrinous arthritis, lung abscesses
2002	120 days	TB	Stifle joint; femoral bone abscess	No antibiotic therapy—owner elected euthanasia	Lung and bone abscesses	1 day	Euthanasia. Right stifle: severe, subacute septic arthritis with abscess formation. Right distal femur and medial condyle: moderate, focal subacute osteomyelitis
2003	39 days	STB	Coxofemoral joint; stifle joint	Amikacin (IV), Ceftiofur (IV), Potassium Penicillin (IV)	Trans-tracheal wash; bone and joint samples culture negative	3 days	Euthanasia. Left coxofemoral joint: severe, chronic necrotizing osteomyelitis with complete necrobiosis of the articular cartilage; pathologic fracture of the acetabular epiphysis. Coxofemoral joints: bilateral chronic septic arthritis
2004	29 days	Shire	Glenohumeral joint; tarsocrural joint	Ceftiofur (IV), Clarithromycin (PO), Rifampin (PO), Amikacin (IA), Timentin (IA)	*R. equi* fecal culture; bone and joint samples culture negative	5 days	Euthanasia. Right distal scapula: severe, acute, focal osteomyelitis and arthritis
2006	90 days	STB	Scapular osteomyelitis	Azithromycin (PO), Rifampin (PO) Amikacin (wound flush), Erythromycin (wound flush)	Shoulder abscess	30 days	Euthanasia. Left scapula: Severe, chronic, locally extensive purulent to necrotizing osteomyelitis with abscessation
2018	90 days	STB	Radiocarpal joint	Chloramphenicol (PO), Clarithromycin (PO), Potassium Penicillin (IV), Rifampin (PO), Amikacin (IA, IV, RLP), Imipenem (IA), Vancomycin (RLP), Piperacillin/Tazobactam (IA, RLP)	Synovial fluid	20 days	Under treatment^*^
2018	90 days	STB	Tarsocrural joint/tibial physis	Clarithromycin (PO), Rifampin (PO) Imipenem (IA), Vancomycin (IO, RLP), Piperacillin/Tazobactam (IA, RLP)	Bone sample and synovial fluid	19 days	Euthanasia. Pathologic tibial fracture

* *Foal discharged from the hospital while under continued treatment and given a poor prognosis for future athletic soundness. TB, Thoroughbred; STB, Standardbred*.

## Results

Twelve foals met the inclusion criteria. Ages ranged from 29 to 365 days with a median age of 111 days. Four breeds were represented: 5 Thoroughbreds, 4 Standardbreds, 2 Arabians, and 1 Shire. The lameness scores on presentation ranged from 1/5 to 5/5, with a median score of 4/5. Two of the most recent cases are highlighted below (sections Case 1 and Case 2).

### Case 1

A 3-month-old Standardbred filly presented with harsh lung sounds and a markedly swollen right carpus. A grade 4/5 (AAEP lameness scale) partially weight-bearing right forelimb lameness was present at the walk. The foal was from a farm with endemic *R. equi* and had been treated for pneumonia with oral rifampin prior to presentation. Standard lateromedial thoracic radiographs were unremarkable. Synoviocentesis of the middle carpal joint revealed purulent yellow fluid with a nucleated cell count of 487 thousand/μL, 94% degenerate neutrophils and a total protein of 7.0 g/dL. Fluid collected from the antebrachiocarpal joint had the same appearance and a cell count of 333 thousand/μL, 92% degenerate neutrophils and a total protein of 6.1 g/dL. Synovial fluid was submitted for antimicrobial culture and sensitivity. Carpal radiographs were unremarkable on admission. Under general anesthesia, the antebrachial and middle carpal joints were treated with arthroscopic joint lavage using egress cannulas and rongeurs to remove fibrin, in combination with intra-articular amikacin (125 mg/joint) and intravenous regional limb perfusion (IVRLP) performed with piperacillin/tazobactam [Zosyn (R); 1 g in 20 mL saline] using a tourniquet applied for 30 min. Oral antimicrobial therapy was switched from rifampin to chloramphenicol due to the lack of radiographic evidence of *R. equi* pneumonia on thoracic radiographs and the excellent bone and synovial penetration of chloramphenicol. The foal continued to receive IVRLP with piperacillin/tazobactam and intra-articular infusion with amikacin for 4 days. Due to limited improvement in the foal's clinical progress, arthroscopic debridement and lavage of both dorsal and palmar aspects of the carpal joints was performed on day 5. Preliminary bacterial culture results yielded *R. equi*; therefore, systemic antimicrobials were switched to oral clarithromycin and rifampin per standard protocol.

**Figure 1:**
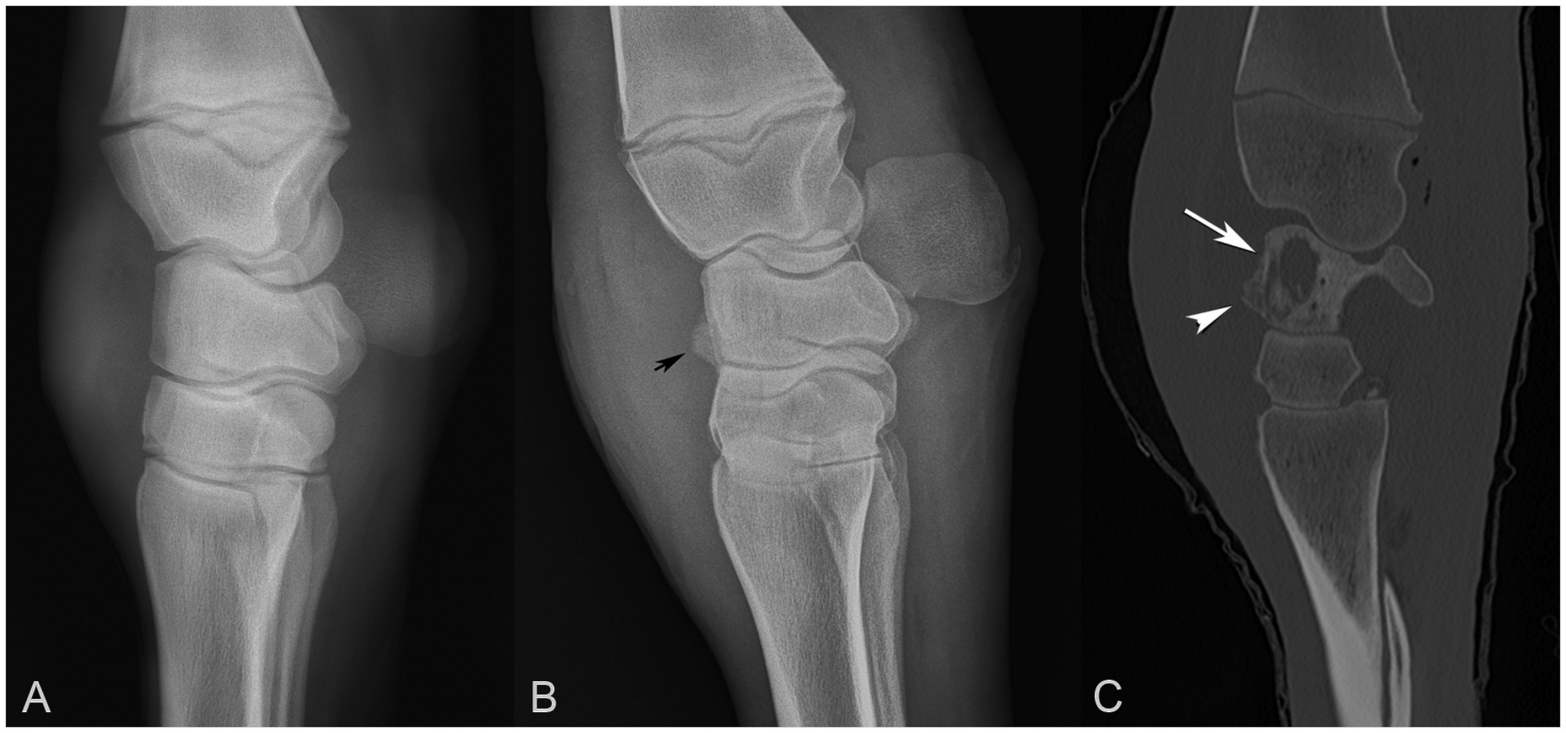
Comparison of sequential radiographs obtained on day 0 and day 18, and bone window computed tomography scan on day 22 (**A–C**, respectively), highlighting the failure of radiographs to elucidate the severity of bone involvement. **(A)** Lateral-medial radiograph taken on day 0. **(B)** Lateral-medial radiograph taken on day 18, revealing osseous proliferation along the dorsal margin of the proximal row of carpal bones (arrow head). **(C)** Sagittal CT image from day 22, revealing lysis of the radiocarpal bone (arrow) with reactive periostitis (arrowhead).

On days 6–10, the foal was sedated for daily joint lavages with isotonic fluids using 14- and 16-gauge needles and infusion of intra-articular antimicrobials. Due to lack of a clinical response, on day 7, piperacillin/tazobactam IVRLP was replaced with vancomycin (250 mg) IVRLP, based on a MIC susceptibility result of ≤ 1.00. On day 8, intra-articular imipenem (25 mg), with a MIC of ≤ 1.00, was added to the treatment regimen to replace amikacin, with a MIC of ≤ 16.00. Despite therapy, the foal's lameness progressed to non-weight bearing. Arthrotomies were performed on day 10, and radiographs performed the same day did not reveal any evidence of osteomyelitis. The foal remained on clarithromycin and rifampin systemically through day 18 of hospitalization. A total of 11 intra-articular infusions were performed; 4 with amikacin and 7 with imipenem. IVRLP was performed every day of hospitalization except on day 18; once with amikacin, 5 times with piperacillin/tazobactam and 11 times with vancomycin. Serial synovial fluid analyses, thoracic radiographs and carpal radiographs were performed throughout hospitalization, with no obvious radiographic changes noted until day 18 (Figure 1).

On day 18, carpal radiographs were repeated, and mild-to-moderate periostitis was noted on the intermediate and third carpal bones, in addition to a questionable focal area of lysis in the radiocarpal bone (Figure 1B). Based on the persistent, severe lameness and index of suspicion of bone involvement radiographically, computed tomography of the carpus and thorax was performed, revealing multiple areas of osteolysis within the carpus. Most significantly affected was the radiocarpal bone, with a 2.4 × 0.7 cm lesion medially, spanning the proximodistal height of the radiocarpal bone (Figure 1C). Smaller lesions were appreciated in the lateral styloid process of the radius, lateral distal subchondral radius and proximolateral third metacarpus. Interestingly, the thoracic cavity had only one small, soft tissue, attenuating nodule in the left caudal lung lobe that was confirmed ultrasonographically as an abscess. The foal was discharged at the owner's request despite a grave prognosis for survival.

### Case 2

A 3-month-old Standardbred filly presented with pneumonia and a swollen left tarsus from the same farm as Case 1. At presentation, the foal was lame at the walk. The foal had been treated with rifampin for 4 days prior to admission. Clarithromycin was added to the systemic antimicrobial regimen upon presentation. Thoracic ultrasonography revealed B-lines and pleural roughening. No *R. equi* pulmonary nodules were appreciated on thoracic radiographs. Synoviocentesis of the left tarsocrural joint yielded turbid joint fluid with a cell count of 3.7 thousand/μL, 21% non-degenerate neutrophils and a total protein of 3.7 g/dL. The foal was anesthetized for joint lavage with egress cannulas, and synovial fluid samples were submitted for culture and sensitivity.

**Figure 2:**
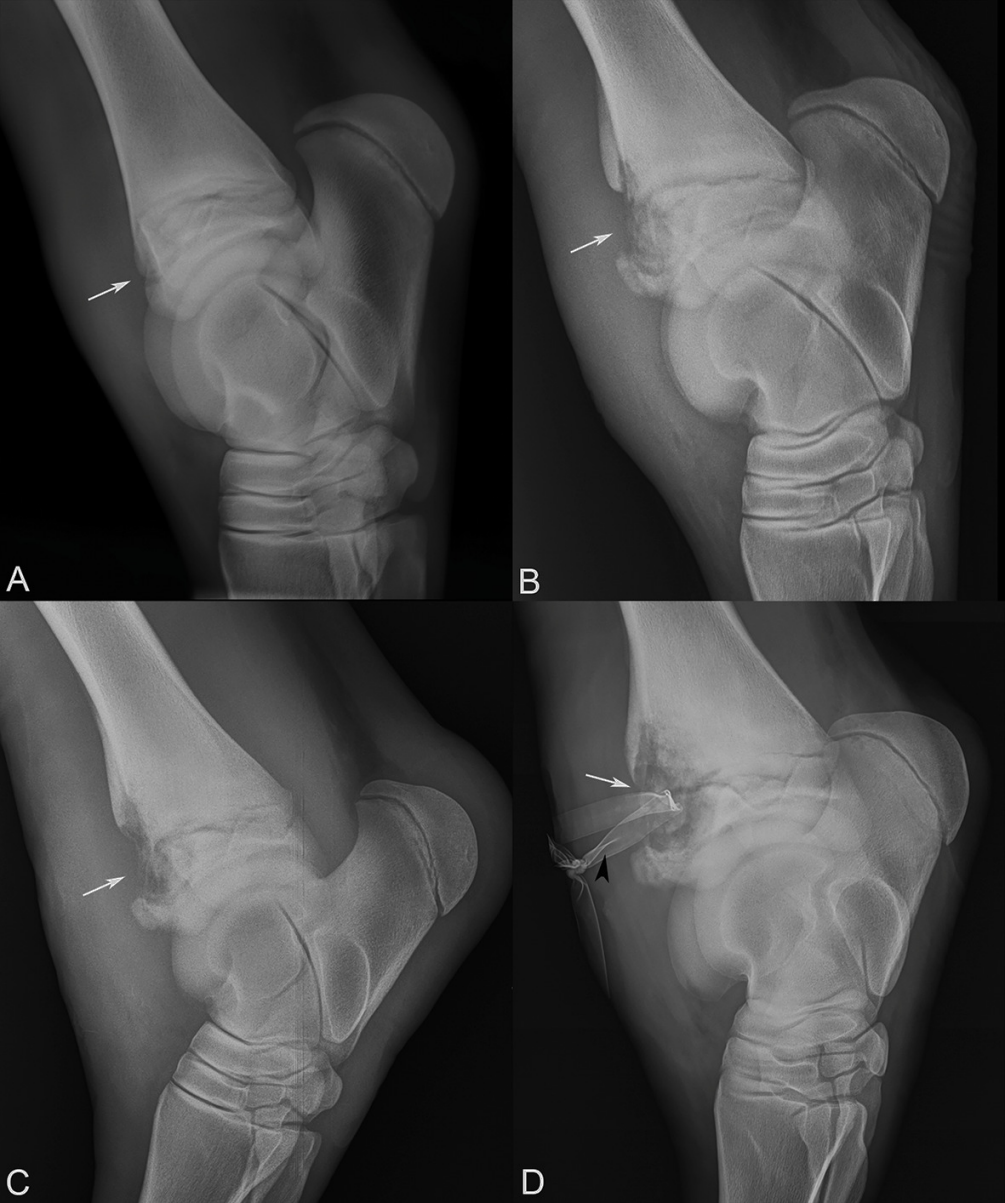
Sequential lateral-medial tarsal radiographs following the progression of the distal tibial physeal lesion (white arrows). **(A)** Referral radiographs, 10 days prior to presentation, **(B)** day 5, **(C)** day 15, and **(D)** day 19 after presentation, revealing severe osteomyelitis and Penrose drain (black arrowhead).

Serial arthrocentesis on days 4, 8 and 11 showed progressively elevated cell counts, increasing from 3.7 thousand/μL to 9.0 thousand/μL, with a marked increase in non-degenerate neutrophil distribution from 21 to 89%; no organisms were ever appreciated on cytologic analysis. Radiographs of the left tarsus revealed a focal, 3.5 cm diameter area of expansion characterized by heterogenous lysis dorsolaterally on the distal tibial physis with moderate periosteal reaction (Figure 2A). There were no radiographic abnormalities of the tarsocrural joint margin or the subchondral bone plate.

Within the first 48 h of hospitalization, the foal received IVRLP with 1 g of piperacillin/tazobactam, and intra-articular injection with 250 mg amikacin after the initial arthrocentesis and subsequent joint lavage. Needle lavage with 1L of isotonic fluid was performed 10 times during hospitalization. Intra-articular antimicrobial infusion following joint lavage consisted of the initial dose of amikacin, and 9 subsequent injections with 25 mg of imipenem. The foal also received daily IVRLP with 1 g of piperacillin/tazobactam from days 1 to 5, followed by 125–250 mg of vancomycin for IVRLP on days 6–17, based on sensitivity results.

The foal was anesthetized 5 times during hospitalization for tarsocrural joint lavage with egress cannulas and large-gauge needles using 5 L of isotonic fluids each time. At the second anesthetic procedure performed on day 6 of hospitalization, a 3.5 mm drill bit was used to allow access to the physis using ultrasonographic and radiographic guidance. A 13-gauge Jamshidi needle was inserted, and a core bone sample was collected and submitted for culture. Vancomycin (125 mg) was injected intra-lesionally through the Jamshidi needle stylet. Imipenem (25 mg) was instilled intra-articularly, and the foal received IVRLP with 125–250 mg vancomycin at the end of each surgery. Intra-osseous injection with a Jamshidi needle and without a tourniquet was repeated on days 8 and day 9. On the days that the foal received IVRLP and intra-lesional injection, the 250 mg vancomycin dose was split −125 mg for IVRLP and 125 mg intra-lesionally. On day 15, a subcutaneous abscess was identified contiguous with the lateral distal tibial physis ultrasonographically. At the fourth surgery, the abscess was incised to evacuate purulent material, followed by lavage and aggressive curettage. A Penrose drain was placed to allow for continued drainage.

In total, the foal was managed for the septic physitis with the development of subcutaneous abscessation and severe synovitis of the left tarsus for 19 days. The physeal lucency continued to expand in size radiographically (Figure 2). On day 19, the lameness became acutely more severe, and radiographs revealed extension of physeal infection and osteomyelitis (Figure 2D). The patient was subsequently euthanized.

### Cases 3–12

The 10 remaining cases presented with lameness, and accompanying swelling and heat of various joints. Lameness scores on admission ranged from 3/5 to 5/5, with a median lameness score of 4/5. Joints involved included the tarsocrural joint (3 cases), stifle joint (3 cases), coxofemoral joint (2 cases), radiocarpal joint (1 case), cubital joint (1 case), glenohumeral joint (1 case) and coffin joint (1 case). Additionally, there were four cases of osteomyelitis/physitis including femoral osteomyelitis with abscessation (1 case), scapular osteomyelitis (2 cases), and distal tibial physitis (1 case).

In 90% (9/10) of cases, radiographs of the affected limb were taken on admission, though only 4 of 9 cases had detectable radiographic osseous lesions. One case was euthanized on admission due to a grave prognosis associated with a large bony abscess involving the right distal femur and medial condyle. Of the remaining nine cases that were admitted, all were started on a combination of antimicrobials including a macrolide plus rifampin and/or ceftiofur. The most common combination was azithromycin and rifampin, which was immediately initiated in 3/11 cases (27%) based on a high index of suspicion of *R. equi*. Culture samples were submitted from a variety of tissues; *R. equi* organisms were positively identified in bone (2/2), synovial fluid (2/8), abscess fluid/material (3/4), trans-tracheal washes (2/2), feces (1/1) and lung tissue (1/1 at necropsy). Synovial fluid samples were typically cultured in aerobic and anaerobic blood culture vials.

Of nine patients that underwent surgical procedures, six had joint lavages performed either through large gauge needles or arthrotomies. The following antimicrobials were instilled intra-articularly: imipenem (3/6), amikacin (5/6), ticarcillin/clavulanic acid (3/6), and erythromycin (1/6). The three patients without joint involvement underwent anesthesia for drainage and debridement of bone abscesses. In eight of the nine patients, euthanasia was elected due to clinical progression of lameness and radiographic progression of lysis and osteoarthritic changes. The mean duration of hospitalization prior to euthanasia was 10.5 days. The ninth patient was found deceased in the stall due to complications from pneumonia.

The only patient discharged alive presented with a left forelimb lameness with heat and swelling localized to the coffin joint. The 45-day old foal arrived from a *Rhodococcus*-endemic farm. Distal limb radiographs did not reveal any bony abnormalities, but thoracic films showed focal pulmonary radiopacities consistent with abscessation. Based on pulmonary lesions and a coffin joint synovial fluid total nucleated cell count of 600 thousand/μL with gram-positive bacteria identified, a diagnosis of *R. equi* was presumed, but not able to be confirmed on synovial fluid culture. The foal was treated with gentamicin and potassium penicillin broad-spectrum antimicrobial therapy for the first 24 h of admission, then switched to gentamicin and azithromycin for the remainder of the hospitalization. In addition, the joint was lavaged, and amikacin was infused intra-articularly on days 1, 2, and 7 of hospitalization. Serial synovial fluid arthrocenteses improved over the course of a 15-day hospital stay, and the foal was discharged on day 15 and lost to follow up.

## Discussion

In the population of foals presenting to the Cornell University Equine Hospital from 1998 to 2018, the prognosis with confirmed *R. equi* joint sepsis or osteomyelitis was poor-to-grave (10/12 euthanized). Limitations of this study include the extended time frame (20 years) over which samples were collected; however, the low frequency of cases with confirmed *R. equi* joint sepsis necessitated this study duration. Furthermore, this case series is limited to a single geographic region, which may not be representative of the entire U.S. However, this may highlight the fact that *R. equi* isolates in different geographic regions may demonstrate variation in antimicrobial susceptibility and/or response to treatment (11). The availability of new antimicrobials such as carabapenems and the increased frequency of IVRLP over other routes of antimicrobial administration represent changes in case management over the timeline of this study.

*R. equi*, a facultative intracellular organism, is challenging to treat due to limited antimicrobial penetration intracellularly and impaired vascular supply to established locations of *R. equi* bone infection in foals (2, 5, 12). In addition, antimicrobial resistant strains of *R. equi* are increasing, potentially due to the widespread use of macrolides and rifampin for sub-clinical disease on *R. equi* endemic farms (5, 10). The most common antimicrobials used to treat *R. equi* based on current therapeutic recommendations include clarithromycin and rifampin (11, 13). This combination of drugs is recommended due to the low minimum inhibitory concentration (MIC), good tissue and macrophage penetration of the drugs and benefit of decreased resistance as a combinatory treatment (14). In the current case series, horses with musculoskeletal involvement unresponsive to initial therapies with rifampin and a macrolide were treated based on culture and susceptibility results, where available. In cases that were refractory to treatment to conventional antimicrobials, antimicrobial selection included amikacin, imipenem, and vancomycin. Data on reported intracellular and intraosseous penetration of specific antimicrobials is variable. Rifampin and certain macrolides, such as clarithromycin, are reported to have excellent intracellular penetration, especially for macrophages, and variable-to-high osseous penetration (14). Limited data is available on osseous penetration for aminoglycosides, carbapenems and glycopeptides; however, these classes of antimicrobials are reported to have strong clinical efficacy against resistant organisms (14).

The distribution of septic joints in the current series reflects that reported in the literature, with the tarsocrural joint most commonly affected (*n* = 4), followed by the stifle (*n* = 3), coxofemoral joint (*n* = 2), radiocarpal joint (*n* = 1), cubital joint (*n* = 1) and glenohumoral joint (*n* = 1). Review of the literature revealed 6 case reports describing 7 foals admitted to tertiary referral centers with an extrapulmonary diagnosis of *R. equi* infection (3–8) and 3 larger case series on *R. equi* in foals (3, 6, 15). The 7 foals presented with primary lameness complaints, all of which were diagnosed with *R. equi* septic synovitis (3/7) and/or osteomyelitis/physitis (6/7) based on positive culture results. Of the 7 foals, 3 were euthanized and 4 were sound at long-term follow up. Septic physitis/osteomyelitis was the most common diagnosis (6/7), which was reported in vertebral bodies, the proximal radius, distal femur, distal pastern, and the distal MT3 physis in two cases. The 4 foals that survived in the above literature reports were diagnosed and treated for septic synovitis with septic physitis (2/4), septic metaphysitis without septic synovitis (1/4) and osteomyelitis without septic synovitis (1/4). Foals that survived were less likely to demonstrate communication between the infected physis and the joint (1/4) (8, 9, 16, 17).

Factors critical to treatment success in prior case series have included institution of aggressive surgical and early medical intervention (3, 6, 15). In 2009, Reuss et al. described *R. equi* extrapulmonary disease in which 111 of 150 (74%) cases diagnosed with *R. equi* pneumonia had at least one extrapulmonary disease (6). Of these cases, nearly 10% were diagnosed with septic synovitis and 3% were diagnosed with osteomyelitis (6). While the reported prognosis for *R. equi*-associated non-septic synovitis, physitis, or osteomyelitis is fair-to-good in the majority of reports (8, 9, 17), the prognosis is generally poor for *R. equi-*associated septic synovitis (6, 15, 18, 19). In both the literature and the current case series, the prognosis appears to be poorest for cases with confirmed septic arthritis as compared to osteomyelitis without septic arthritis.

Advanced imaging, such as MRI, nuclear scintigraphy or computed tomography, may have led to earlier detection of the extent of bone and joint involvement in these cases. Notably, bone involvement was not detected on carpal radiographs in Case 1 until later in the course of disease, and computed tomography may have had an increased sensitivity for detection of early osseous changes. A proposed change in management protocol may include computed tomography in all cases presenting for lameness secondary to *R. equi* septic arthritis, osteomyelitis, physitis or for cases diagnosed with septic arthritis and coming from a farm endemic for *R. equi*. When bone involvement is confirmed, aggressive local therapies including joint lavage, surgical debridement, and local antimicrobial delivery should be pursued, but may still be insufficient to resolve the infection without a more targeted delivery and increased efficacy of antimicrobials. Because of challenges associated with antimicrobial penetration into necrotic bone and joint tissues, alternative non-antimicrobial strategies may also need to be considered. In the two cases described in detail, pathologic sequelae and failure to resolve the infection despite aggressive therapy, including repeated intra-articular lavage; arthroscopy; arthrotomies; bone debridement and curettage; and regional, intra-articular, intra-osseous and systemic antimicrobials highlight the resilience of the organism.

## Conclusion

The first 2 case studies provide excellent examples of some of the challenges and complications that can be associated with treatment of *R. equi* orthopedic infections and highlight the failure of aggressive antimicrobial regimens, including glycopeptides and carbapenems, to resolve these infections. These cases highlight the need for novel therapeutic approaches to treat *R. equi* orthopedic infection. Future treatments may include novel antimicrobials or non-traditional delivery modalities, such as liposomal formulations of gentamicin (5). Preventative approaches for decreasing the prevalence of endemic *R. equi* should be a continued priority, including research into vaccinations (20). In the meantime, the use of prophylactic antibiotics needs to be weighed against the increase in resistance (12). More aggressive initial diagnostic approaches may be required for *R. equi* orthopedic infection, including the use of nuclear scintigraphy, CT or MRI. Despite aggressive surgical intervention, *R. equi* joint infection is associated with a grave prognosis for athletic activity and a poor prognosis for survival in foals, and efforts should focus on both preventing infection and developing more effective antimicrobial regimens to target *R. equi*.

## Data Availability Statement

All data analyzed for this case series are included within the manuscript.

## Author Contributions

NR performed the medical records search. NR, LL, and HR drafted and edited the first versions of the manuscript. LL, HR, LF, and ND were involved in diagnostic and therapeutic case management of Cases 1 and 2. All authors edited and approved the final version of the manuscript.

### Conflict of Interest

The authors declare that the research was conducted in the absence of any commercial or financial relationships that could be construed as a potential conflict of interest.
